# Supplementary material for: INT-767—A Dual Farnesoid-X Receptor (FXR) and Takeda G Protein-Coupled Receptor-5 (TGR5) Agonist Improves Survival in Rats and Attenuates Intestinal Ischemia Reperfusion Injury
Source: Int J Mol Sci. 2023 Oct 4;24(19):14881. doi: 10.3390/ijms241914881 (PMC10573246; doi:10.3390/ijms241914881)
Supplement: Supplementary file 1 [file ijms-24-14881-s001.zip › Supplementary file S1.pdf]

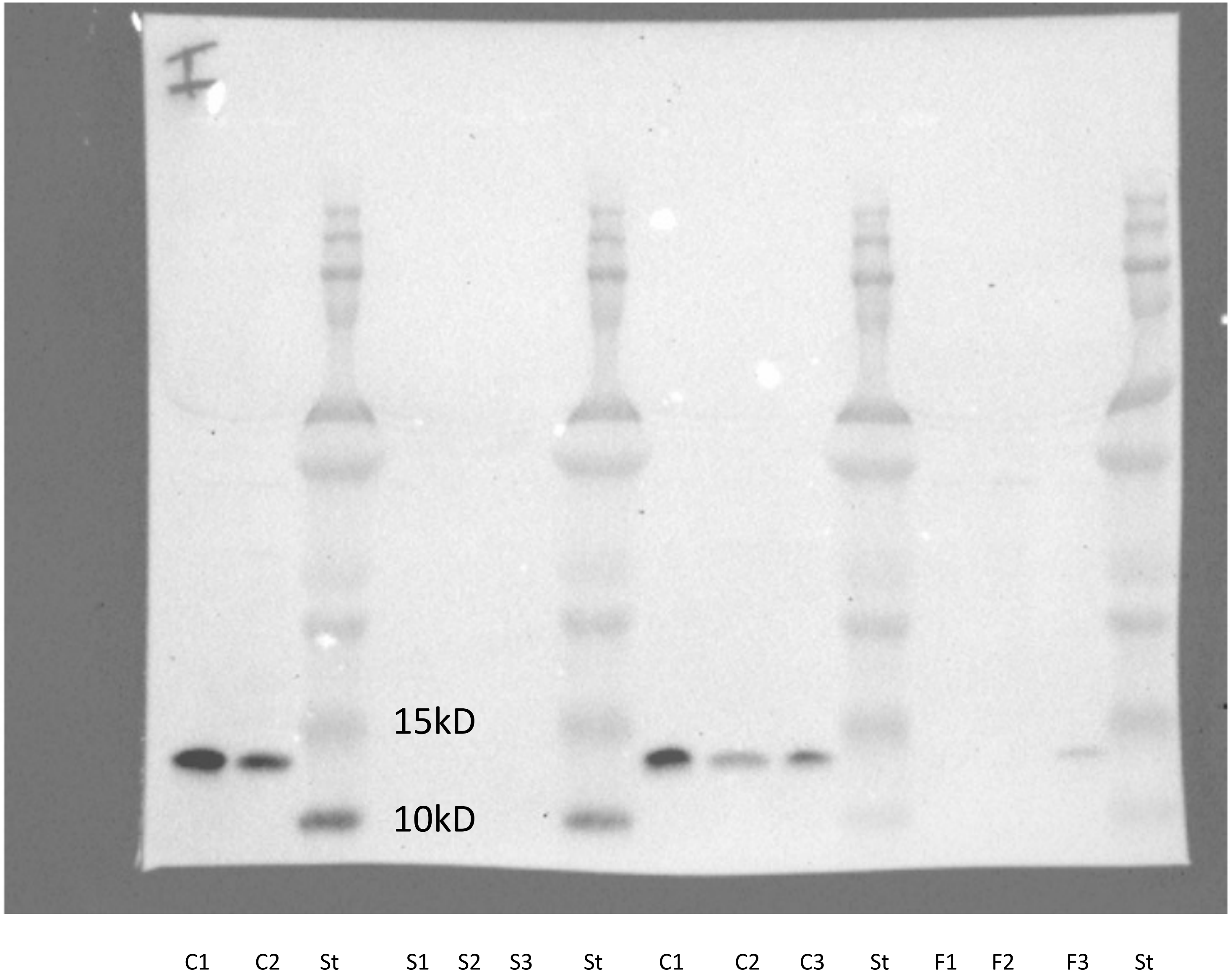

Legend:

C = control

S = sham

F = FXR

St = standard

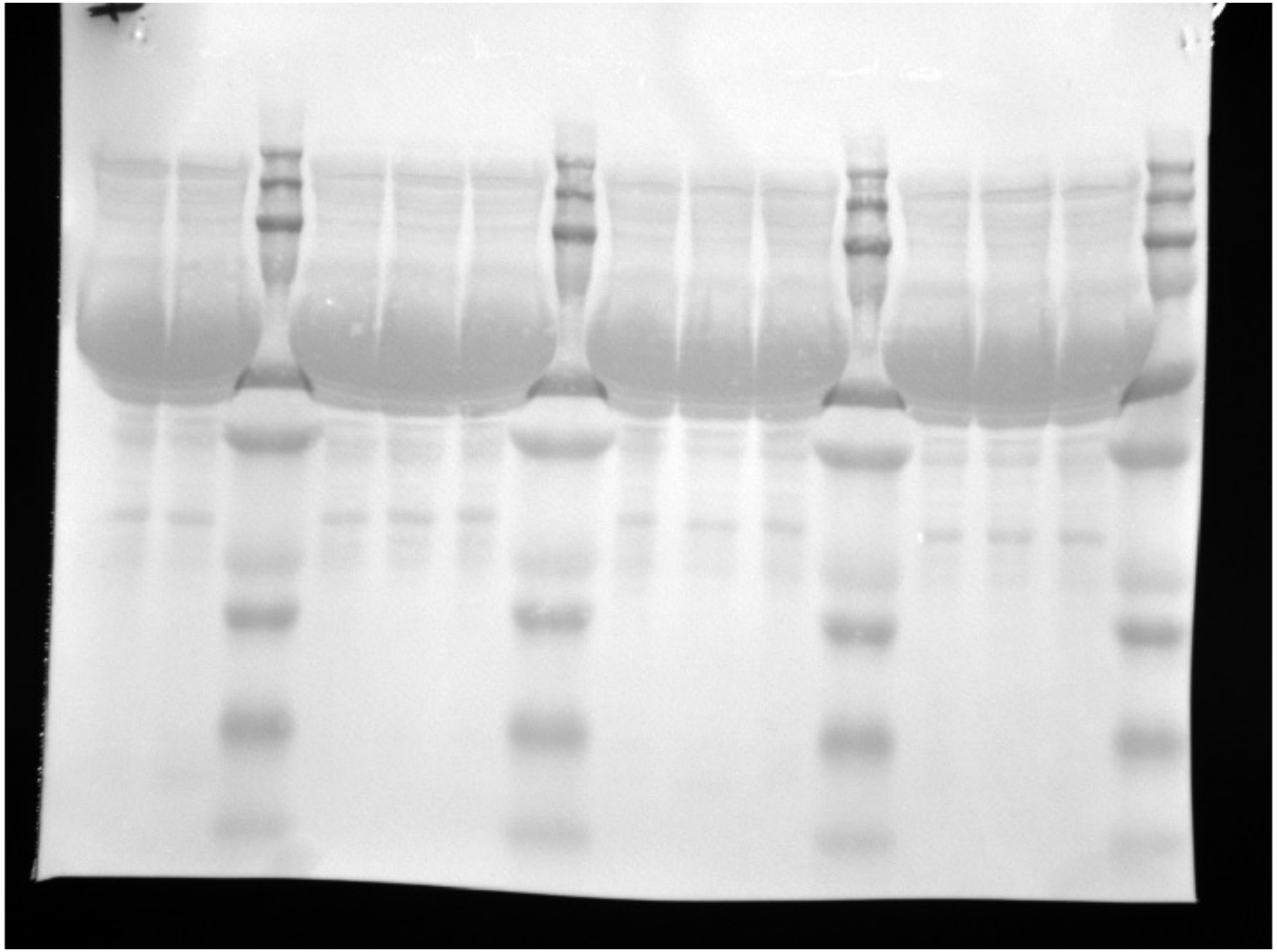

C1 C2 St S1 S2 S3 St C1 C2 C3 St F1 F2 F3 St

Ponceau S staining

Blot 1

C = control

S = sham

F = FXR

St = standard

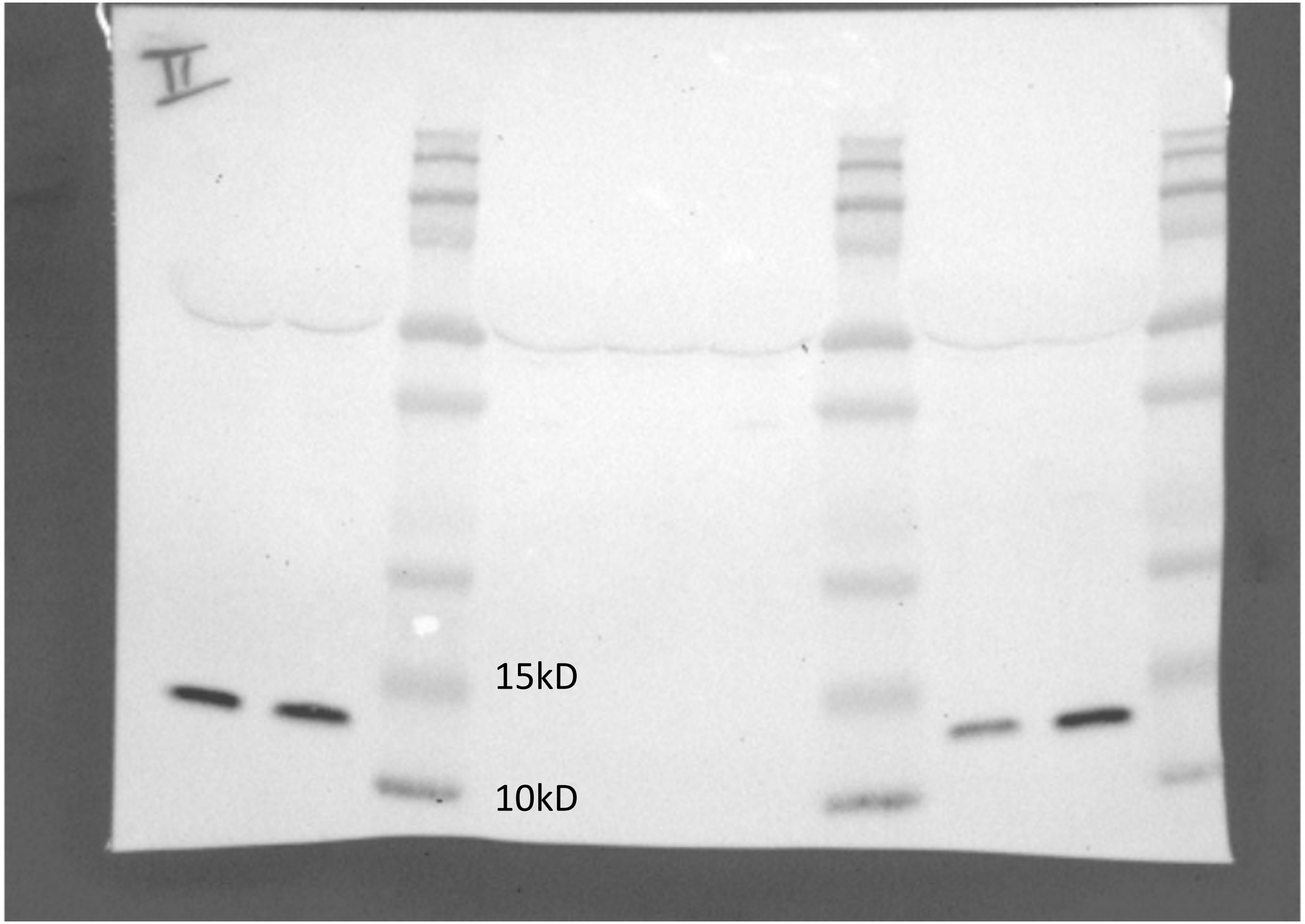

C1 C2 St S4 S5 S6 St C4 C5. St

Legend:

C = control

S = sham

F = FXR

St = standard

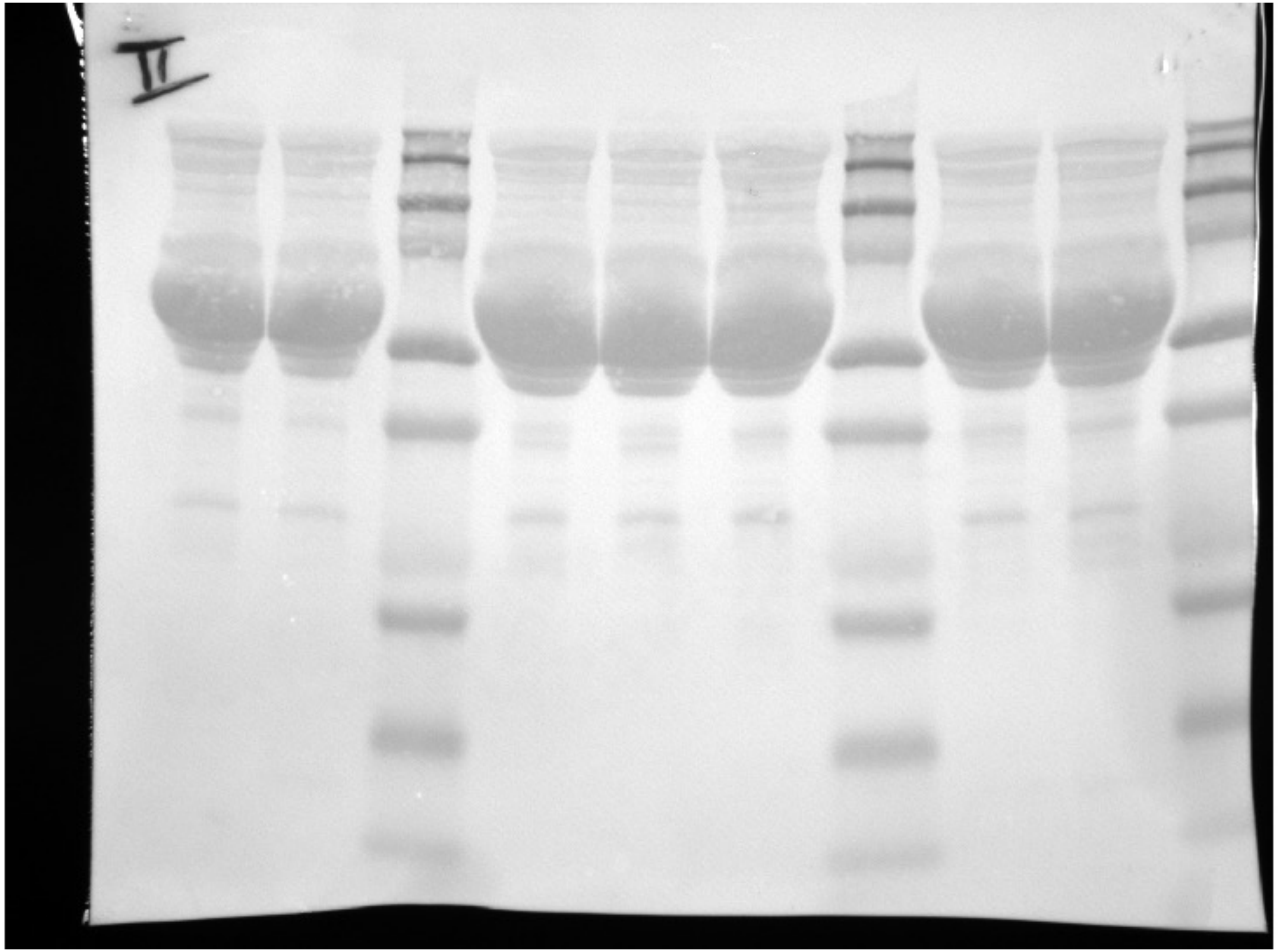

C1 C2 St S4 S5 S6 St C4 C5. St

Ponceau S staining

Blot 2

C = control

S = sham

F = FXR

St = standard

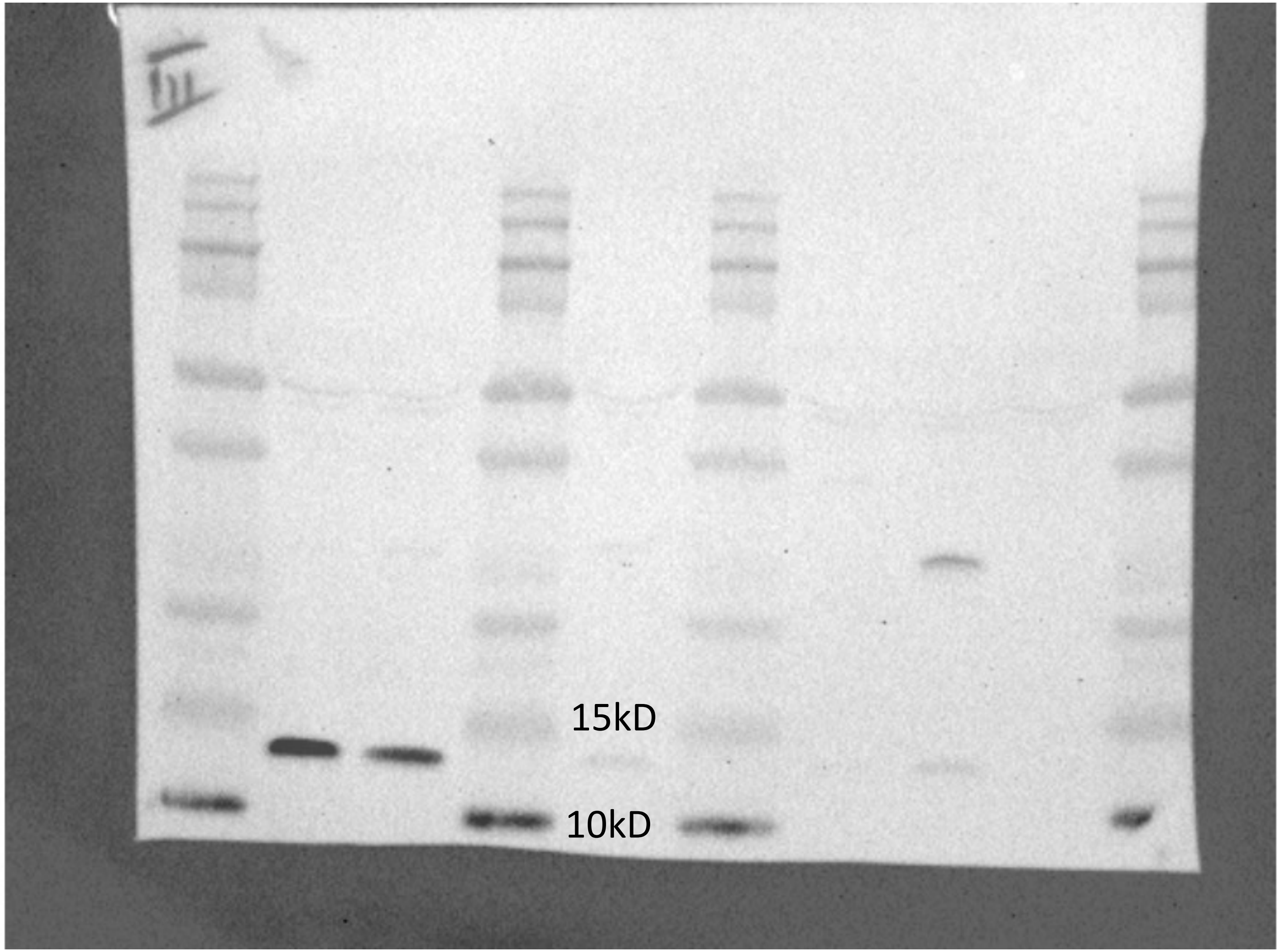

St C1 C2 St C6 St F4 F5 F6 St

Legend:

C = control

S = sham

F = FXR

St = standard

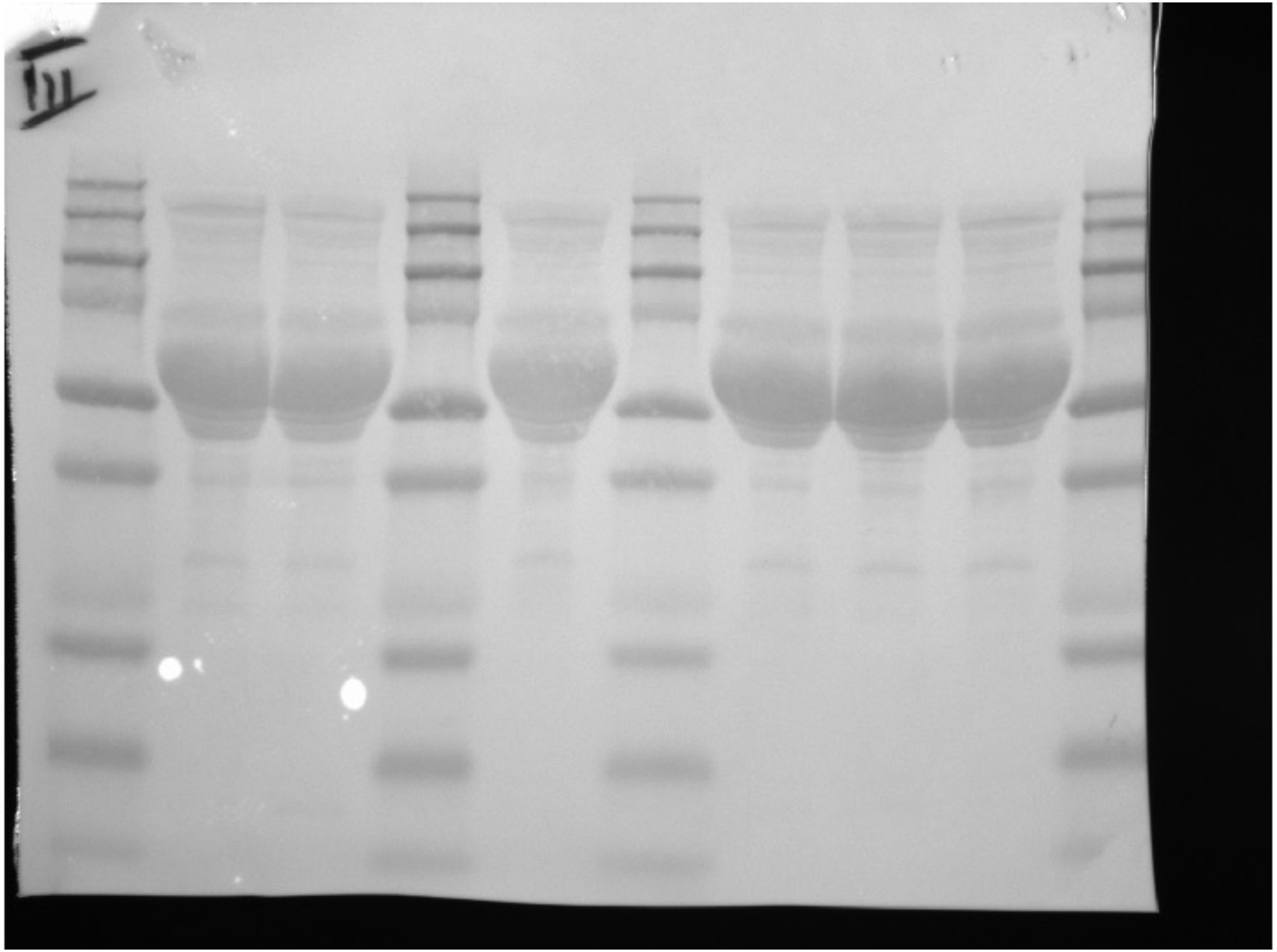

Ponceau S staining

Blot 3

C = control

S = sham

F = FXR

St = standard
