# Supplementary material for: INT-767—A Dual Farnesoid-X Receptor (FXR) and Takeda G Protein-Coupled Receptor-5 (TGR5) Agonist Improves Survival in Rats and Attenuates Intestinal Ischemia Reperfusion Injury
Source: Int J Mol Sci. 2023 Oct 4;24(19):14881. doi: 10.3390/ijms241914881 (PMC10573246; doi:10.3390/ijms241914881)
Supplement: Supplementary file 1 [file ijms-24-14881-s001.zip › Supplementary File S2 TGR5.pdf]

TGR5

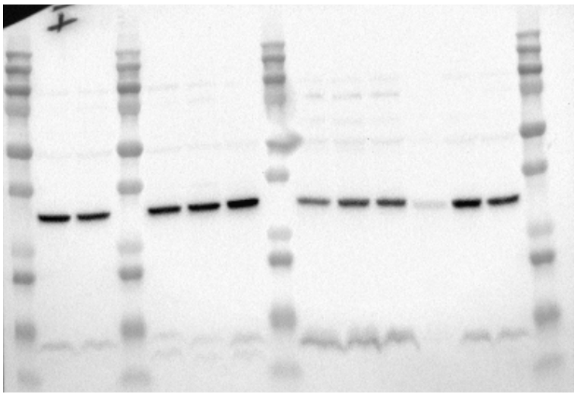

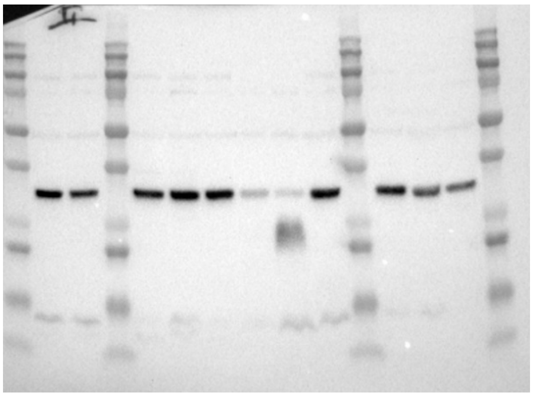

St-F2-F3-St-S1-S2-S3-St-C1-C2-C3-F1-F2-F3-St   St-F2-F3-St-S4-S5-S6-C4-C5-C6-St-F4-F5-F6-St

Legend:

C= Control

S= Sham

F = FXR (treatment group)

St = Standard

B-Actin

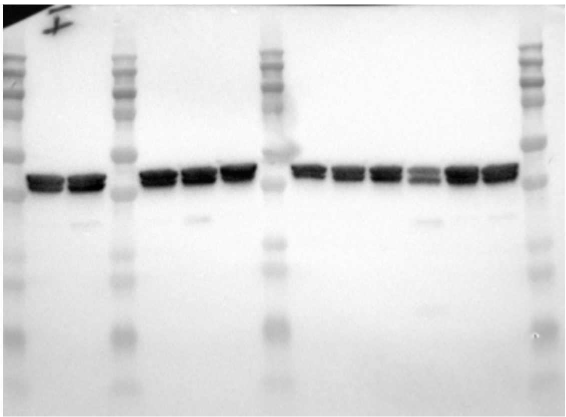

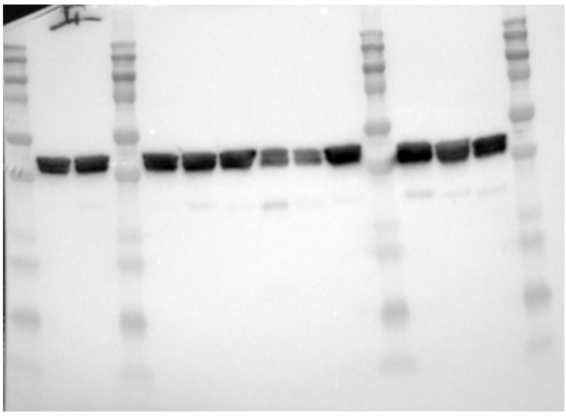

St-F2-F3-St-S1-S2-S3-St-C1-C2-C3-F1-F2-F3-St   St-F2-F3-St-S4-S5-S6-C4-C5-C6-St-F4-F5-F6-St
