# Supplementary material for: INT-767—A Dual Farnesoid-X Receptor (FXR) and Takeda G Protein-Coupled Receptor-5 (TGR5) Agonist Improves Survival in Rats and Attenuates Intestinal Ischemia Reperfusion Injury
Source: Int J Mol Sci. 2023 Oct 4;24(19):14881. doi: 10.3390/ijms241914881 (PMC10573246; doi:10.3390/ijms241914881)
Supplement: Supplementary file 1 [file ijms-24-14881-s001.zip › Supplementary file S3 Power calculations.pdf]

Supplementary file S2 – Sample size power calculation

The rationale for the number in each group was based on power calculation which were based on predictions from the previous study from this model[1].

The primary end-point chosen was the level of histological damage, calculated by the Park-Chiu score (0-8). The expected Park-Chiu score for histological damage (range: 0-8) was 5 (±1.5) in the control group versus 2.5 in the treated group in our previous study. This resulted in a calculated group size of 6 (continuous endpoint, with an alpha of 0.05 and power of 80%).

$$k = \frac{n_2}{n_1} = 1$$

$$n_1 = \frac{(\sigma_1^2 + \sigma_2^2/K)(z_{1-\alpha/2} + z_{1-\beta})^2}{\Delta^2}$$

$$n_1 = \frac{(1.5^2 + 1.5^2/1)(1.96 + 0.84)^2}{2.5^2}$$

$$n_1 = 6$$

$$n_2 = K * n_1 = 6$$

$\Delta = |\mu_2 - \mu_1|$ = absolute difference between two means
σ_1, σ_2 = variance of mean #1 and #2
n_1 = sample size for group #1
n_2 = sample size for group #2
α = probability of type I error (usually 0.05)
β = probability of type II error (usually 0.2)
z = critical Z value for a given α or β
k = ratio of sample size for group #2 to group #1

Calculation were performed using an online sample size calculator:

https://clincalc.com/stats/samplesize.aspx

References:

1. Ceulemans LJ, Verbeke L, Decuypere JP, et al. Farnesoid x receptor activation attenuates intestinal ischemia reperfusion injury in rats. *PLoS One*. 2017;12(1):1-17. doi:10.1371/journal.pone.0169331
